# Supplementary material for: A biomimetic nanoreactor for synergistic chemiexcited photodynamic therapy and starvation therapy against tumor metastasis
Source: Nat Commun. 2018 Nov 28;9:5044. doi: 10.1038/s41467-018-07197-8 (PMC6262009; doi:10.1038/s41467-018-07197-8)
Supplement: Supplementary file 1 — Supplementary Information [file 41467_2018_7197_MOESM1_ESM.pdf]

Supplementary Methods

A biomimetic nanoreactor for synergistic chemiexcited photodynamic therapy and starvation therapy against tumor metastasis

Yu *et al.*

Instruments. High-resolution transmission electron microscope (HRTEM, JEM-2100). Fluorescence spectra were obtained with an Edinburgh fluorescence spectrometer with a xenon lamp (FLS-920). Absorption spectra were measured on a pharmaspec UV-1700 UV-visible spectrophotometer (Shimadzu, Japan). All pH measurements were made using a pH-3c digital pH meter (Shanghai Leici Equipment Engineering Co., Ltd., Shanghai, China). In the MTT assay, the absorbance was measured using a microplate reader (Synergy 2, Biotek, USA). Cell disruption was performed using a homogenizer (IKA, Germany, T10 basic ultra-turrrax). Confocal fluorescence images were captured using TCS-SP8 confocal laser scanning microscope (Leica, Germany), objective lens (×40, ×60). Elemental analysis was performed using a coupled plasma spectrometer (Thermo Fisher, iCAP 7400, USA). *In vivo* fluorescence images were captured using Bio-Rad imaging system (Bio-Rad, Hercules, CA, USA).

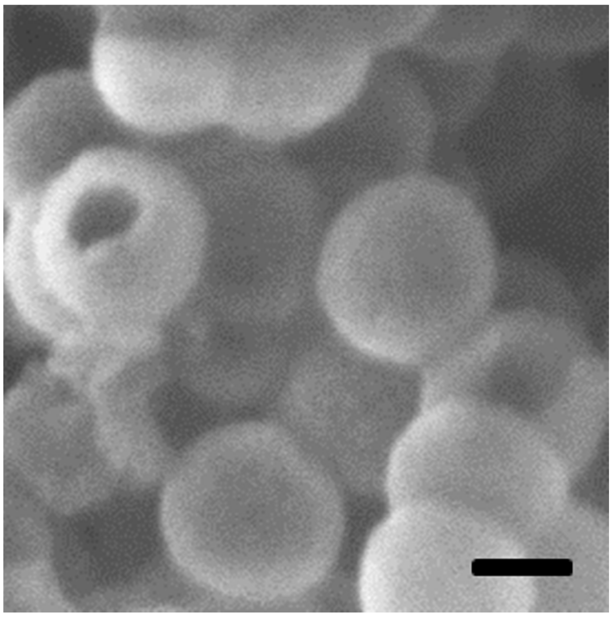

Supplementary Figure 1 SEM image of HMSNs. Scale bar is 50 nm.

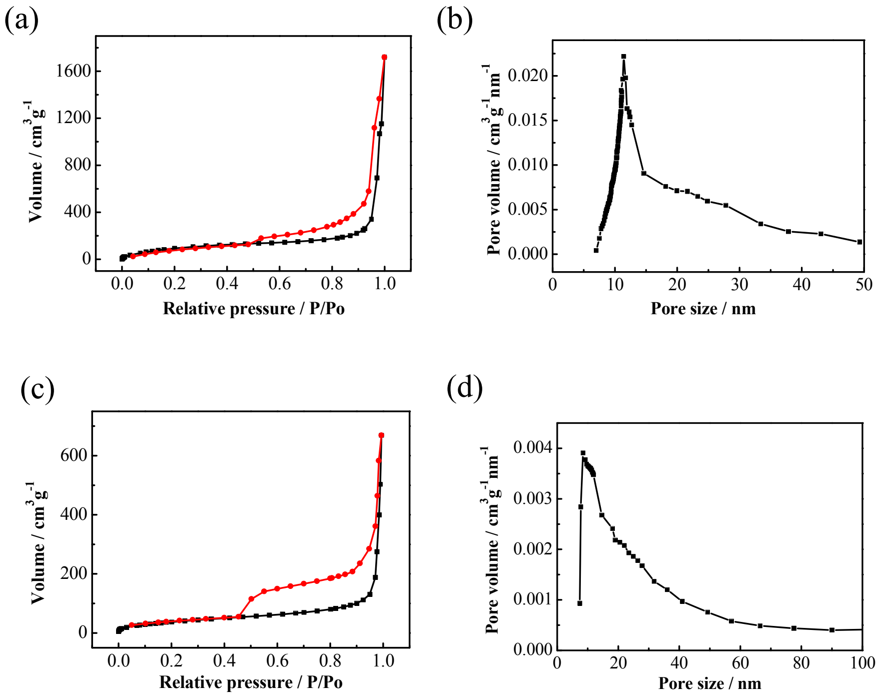

Supplementary Figure 2 N_2 adsorption-desorption isotherms of HMSNs (a) and HMSNs-NH_2 (c). Pore size distribution of HMSNs (b) and HMSNs-NH_2 (d).

Supplementary Table 1. BET surface area and average pore size of HMSNs and HMSNs-NH_2.

	**HMSNs**	**HMSNs-NH_2**
**BET surface area (m^2/g)**	**417.17**	**147.17**
**Pore size (nm)**	**11.4**	**8.43**

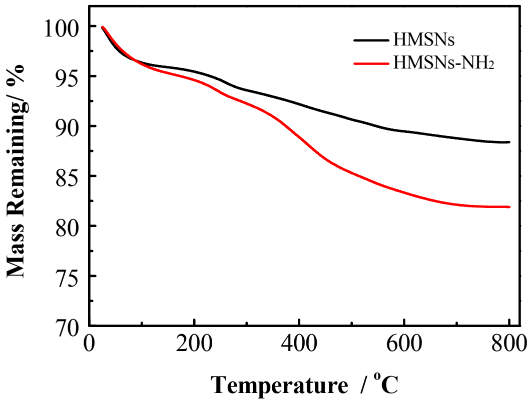

Supplementary Figure 3 TGA analysis of HMSNs and HMSNs-NH_2.

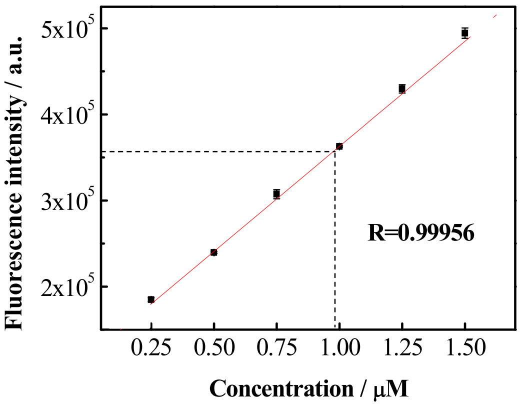

Supplementary Figure 4 Standard linear calibration curve of Ce6.

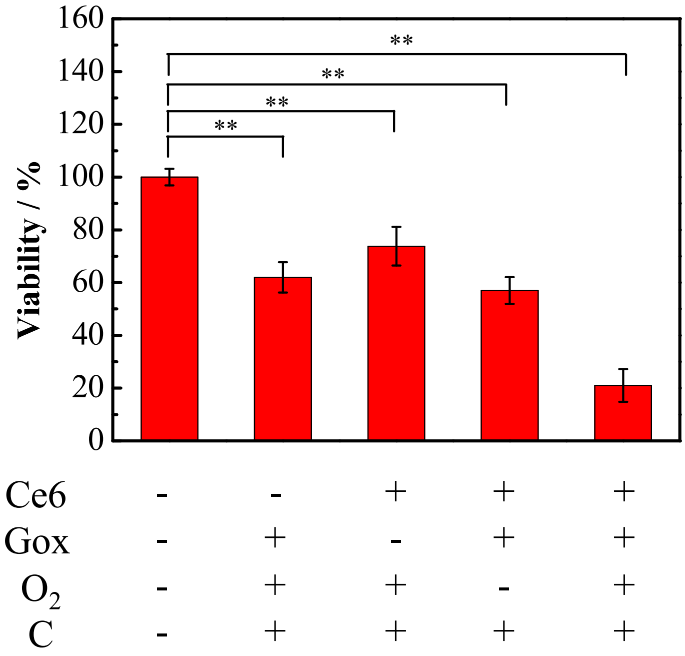

Supplementary Figure 5 MTT assay of B16-F10 cells with different treatments in the hypoxia environment (student's t-test, **p< 0.01).

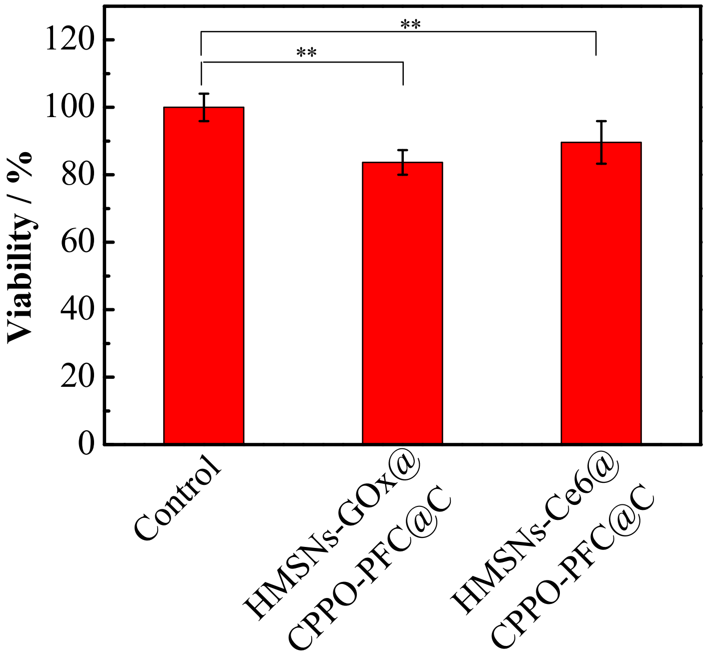

Supplementary Figure 6 Cell viability after starvation therapy alone or PDT alone in the anaerobic conditions (student's t-test, **$p < 0.01$).

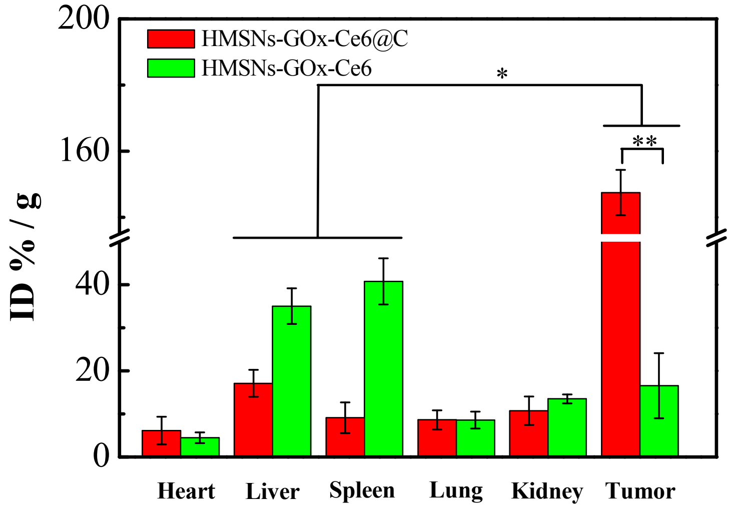

Supplementary Figure 7 Si content in different organs and metastatic tumors after the mice were injected with HMSNs-GOx-Ce6@C or HMSNs-GOx-Ce6 *via* ICP-AES analysis (student's t-test, *$p < 0.05$, **$p < 0.01$).

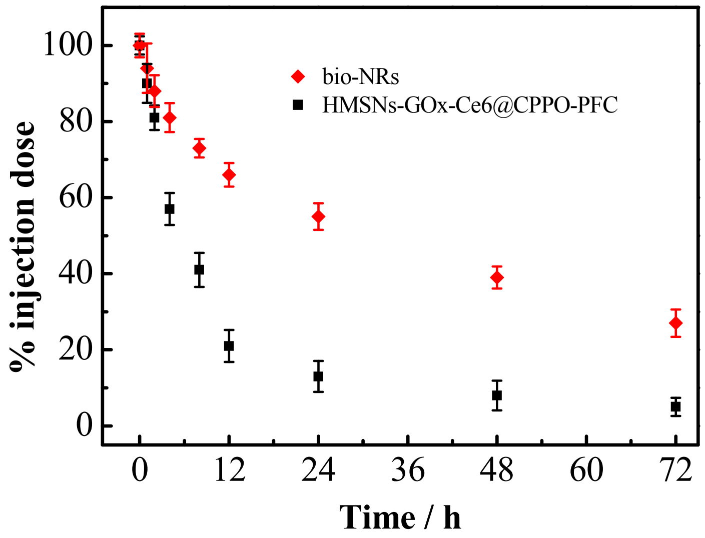

Supplementary Figure 8 Retention of the bio-NRs and HMSNs-GOx-Ce6@CPPO-PFC in the body at different time via ICP-AES analysis.

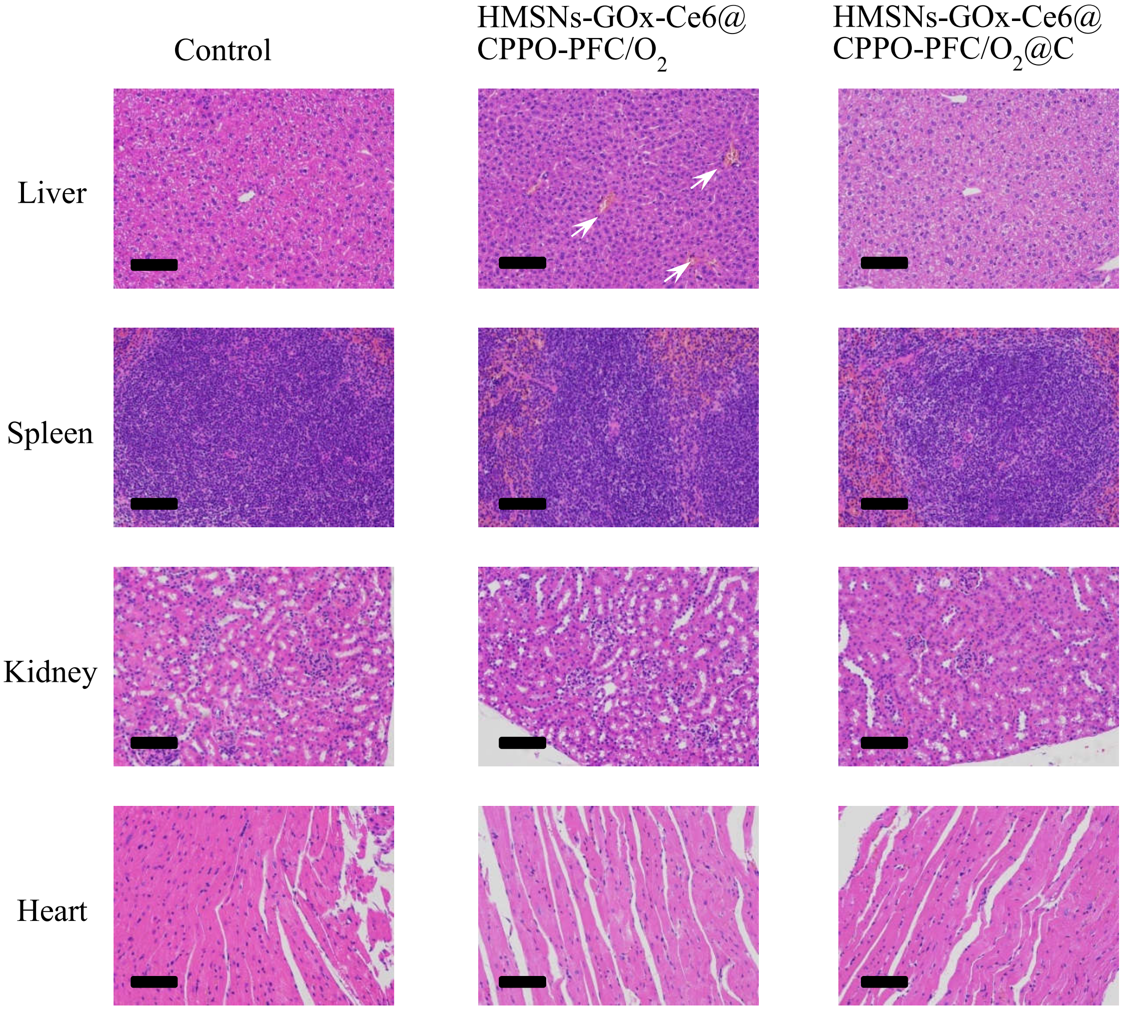

Supplementary Figure 9 H&E staining images of four major organs (liver, spleen, kidney, and heart) after the mice were treated with PBS, HMSNs-GOx-Ce6@CPPO-PFC/O_2 or bio-NRs (200×, scale bars are 100 μm.). The arrows are referring to liver injury.
